# Shorter but Better? A Cross-Cultural Analysis of Sleep in Japan and the U.S. Using

**DOI:** 10.1093/geroni/igaf122.769

**Published:** 2025-12-31

**Authors:** Kyoungmin Cho, Soomi Lee

**Affiliations:** The Pennsylvania State University, University Park, Pennsylvania, United States; The Pennsylvania State University, University Park, Pennsylvania, United States

## Abstract

Poor sleep is an increasing global concern, yet research comparing cross-country differences in sleep quality remains limited. This study examined cross-cultural differences in sleep patterns between Japan and the United States. We analyzed two-wave data from 618 adults who participated in the Midlife in the United States (MIDUS; Wave1=2004–2006, Wave2=2013–2014, n = 535, M_age=51.6) and Midlife in Japan (MIDJA; Wave1=2008, Wave2=2012, n = 83, M_age=50.5). Sleep was commonly assessed using the Pittsburgh Sleep Quality Index, a well-validated measure assessing habitual sleep quality, sleep latency, sleep duration, habitual sleep efficiency, sleep disturbance, sleeping medication use, and daytime dysfunction (PSQI; Range=0-21; the higher score, the better). In the ANCOVA, baseline age, gender, education, and self-rated health were controlled for. At Wave1, Japanese adults showed better global PSQI scores compared to US adults (F(1,611)=20.80, p<.001, Mean_US =5.96 vs. Mean_Japan=4.08). They also exhibited shorter sleep latency, higher sleep efficiency, fewer sleep disturbances, and lower medication use. However, sleep duration, sleep quality, and daytime dysfunction did not differ significantly. At Wave2, Japanese adults continued to have better sleep (F(1,611)=14.66, p<.001, Mean_US=6.37 vs. Mean_Japan=4.83). Japanese adults reported shorter sleep latency, fewer sleep disturbances, and lower medication use. However, they slept for a shorter duration than their U.S. counterparts (F(1, 611)=17.94, p<.001, Mean_US=6.91 hrs vs. Mean_Japan=6.27 hrs). Sleep quality, sleep efficiency and daytime dysfunction did not significantly differ at Wave2. Despite shorter duration, Japanese adults reported better sleep quality than U.S. adults. Further research should explore cultural and lifestyle influences in cross-cultural differences in sleep.

